# Frailty identification and management among Brazilian healthcare professionals: a survey

**DOI:** 10.1186/s12877-024-05020-2

**Published:** 2024-06-03

**Authors:** Paula Schmidt Azevedo, Ruth Caldeira de Melo, Juli Thomaz de Souza, Rachael Frost, James P. Gavin, Katie Robinson, Paulo José Fortes Villas Boas, Marcos Ferreira Minicucci, Ivan Aprahamian, Patrick Alexander Wachholz, Kathryn Hinslif-Smith, Adam Lee Gordon

**Affiliations:** 1 Botucatu Medical School, São Paulo State University (Unesp), District of Rubião Junior, no number, Botucatu, SP 18618-970 Brazil; 2 School of Arts, Sciences and Humanities, University of São Paulo, São Paulo, Brazil; 3 Research Department of Primary Care and Population Health, University College London, London, UK; 4 School of Health Sciences, University of Southampton, Southampton, UK; 5 Academic Unit of Injury, Recovery and Inflammation Sciences (IRIS), University of Nottingham, Nottingham, UK; 6 Faculty of Medicine of Jundiaí, Jundiaí, São Paulo, Brazil; 7 Leicester School of Nursing and Midwifery, Faculty of Health and Life Sciences, De Montfort University, Leicester, UK; 8 NIHR Applied Research Collaboration – East Midlands (ARC-EM), Nottingham, UK

**Keywords:** Frailty, Frailty criteria, Healthcare professionals, Older people, Survey, Brazil

## Abstract

**Background:**

National and international guidelines on frailty assessment and management recommend frailty screening in older people. This study aimed to determine how Brazilian healthcare professionals (HCPs) identify and manage frailty in practice.

**Methods:**

An anonymous online survey on the assessment and management of frailty was circulated virtually through HCPs across Brazil.

**Results:**

Most of the respondants used non-specific criteria such as gait speed (45%), handgrip strength (37.6%), and comprehensive geriatric assessment (33.2%). The use of frailty-specific criteria was lower than 50%. The most frequently used criteria were the Frailty Index (19.1%), Frailty Phenotype (13.2%), and FRAIL (12.5%). Only 43.5% felt confident, and 40% had a plan to manage frailty. In the multivariate-adjusted models, training was the most crucial factor associated with assessing frailty, confidence, and having a management plan (*p* < 0.001 for all). Those with fewer years of experience were more likely to evaluate frailty (*p* = 0.009). Being a doctor increased the chance of using a specific tool; the opposite was true for dietitians (*p* = 0.03). Those who assisted more older people had a higher likelihood of having a plan (*p* = 0.011).

**Conclusion:**

Frailty assessment was heterogeneous among healthcare professions groups, predominantly using non-specific criteria. Training contributed to frailty assessment, use of specific criteria, confidence, and having a management plan. This data informs the need for standardized screening criteria and management plans for frailty, in association with increasing training at the national level for all the HCPs who assist older people.

## Background

Frailty is a complex syndrome defined as *“a clinical state in which there is an increase in an individual’s vulnerability for developing increased dependency and mortality when exposed to a stressor.”* [1] It is associated with adverse outcomes in older people, including falls, hospitalization, dependency, and death [2]. In Brazil, the prevalence of frailty in non-institutionalized older people is estimated at 24%, higher than in more developed countries [3, 4]. Frailty increases in prevalence with age, but its progress can be prevented and reversed [2]. 

International Clinical Practice Guidelines recommend that health professionals screen all adults over 65 for frailty, using valid and rapid frailty instruments that should also be suitable for the setting or context [1]. In Brazil, a task force comprising researchers and professionals specialized in human aging developed a consensus about frailty concepts and identification [5]. The Brazilian consensus highlights the need for every health professional who assists older people to be familiar with the frailty syndrome and its consequences. Identifying pre-frailty and frailty improves the chances of early intervention, enabling early management and prevention of deterioration. Identifying more advanced frailty, meanwhile, enables person-centered anticipatory care in a timely fashion [2]. However, a lack of knowledge leads to a lack of standardization of frailty screening, with a gap between what the guidelines recommend and clinical practice [5]. 

Around 30 criteria have been described that assess frailty, including the Frailty phenotype (from the Cardiovascular Health Study), Frailty Index, Study of Osteoporotic Fractures tool (SOF), Clinical Frailty Scale (CFS), FRAIL Scale, Edmonton Frailty Scale (EFS), PRISMA-7 and the Tilburg Frailty Index (TFI) [5, 6]. In the Brazilian Consensus on Frailty in Older People, the authors found these criteria are already used and validated for Brazilian practice, at least in part. However, researchers frequently adapt previously validated criteria, using non-standard cut points in their studies, further contributing to the need for more standardization [5]. 

Mesquita and Ricci (2022) found that Brazilian primary healthcare professionals reported very little or limited knowledge concerning frailty (52.6%) and demonstrated low practical knowledge (55.1%). Only 12.5% of their respondents could correctly define the frailty syndrome [7]. Strategies are needed to reduce the “know-do gap” around frailty in the care of older Brazilians [8]. However, before developing approaches to improve the situation, we need to understand the current state of knowledge better and practice around frailty amongst Brazilian healthcare professionals (HCPs) across a broader range of settings.

This study aimed to understand how Brazilian HCPs, working in different settings, identify and manage frailty in practice and start exploring the reasons for their responses.

## Methods

This observational cross-sectional study was conducted with HCPs over 18 years using an online questionnaire. All participants who agreed to participate gave consent electronically before completing the online survey. The survey was circulated among HCPs (Medical doctors, nurses, physiotherapists, dietitians, gerontologists, and other health professionals, such as dentists, psychologists, older people caregivers, nursing assistants, and pharmacists) using a snowball technique, where each respondent was asked to share the survey details with colleagues. We aimed to assess the heterogeneity of respondents by clinical background and experience within their profession in caring for older people with frailty. This would maximize the range of responses about their current frailty assessment criteria.

REDCap (Research Electronic Data Capture) software was used to create and manage the online survey data. Piloting of the survey suggested it took 5–10 min to complete all 24 questions.

The survey was developed in partnership with academic researchers from the United Kingdom (UK) as part of an ongoing collaboration around frailty that started in 2018 during the workshop: “Identifying and addressing shared challenges in conducting health and social care research for older people (OPAL)”, funded by British Council, Newton funding and The São Paulo research foundation (FAPESP). The questions were based on the recommendations in guidelines published in both countries [5, 9]. A UK version was first designed and circulated across the UK [10]. Then, the Brazilian team (JTS, MFM) translated the questionnaire into Brazilian Portuguese, adapting some questions and terminologies for the national context. The questionnaire was then back translated into English by a UK native fluent in Portuguese. The UK and BR teams approved the final version. The online survey link was distributed from Aug/2020 to Dec/2021.

The invitation to participate in the study was circulated via social networks, including Facebook and Twitter, researcher networks, and the mailing lists of two public universities in São Paulo State (including all the current employees of clinical hospitals and medical schools alongside alumni including nurses, nurse technicians, dietitians, physiotherapists, social workers, and gerontologists) and the Brazilian Society for Food and Nutrition (SBAN). In Brazil, gerontologists are trained either at the undergraduate or postgraduate level and are non-medical professionals specializing in the care of older people.

The online electronic survey was divided into two parts. Part one included demographic data, including socio-demographic characteristics (sex, age range, and ethnicity); working context (nature of the institution, years and main place spent in clinical practice, experience with caring for older people, and approximate proportion of older adults assisted by day); and then part two focussed on frailty assessment and management.

The second part of the online survey included multiple-choice questions comprising dichotomous and Likert-like questions regarding (a) criteria used to evaluate frailty in practice, (b) frequency of frailty assessment, (c) value of frailty assessment, (d) formal training received to identify frailty, (e) modification of care plan based on frailty level; (f) additional routine assessment after frailty identification; (g) referral pathways; (h) development of specific frailty care plans; (i) confidence in managing frailty and; (j) formal training received to manage frailty. Finally, at the end of the survey, each respondent was invited to an open narrative to include any aspects of frailty management, including their confidence levels for managing frailty.

Regarding the Frailty Assessment, TFI, PRISMA-7, SOF, EFS, CFS, FRAIL, Fried Phenotype, and Frailty Index were considered **specific criteria.** [1, 6] Comprehensive Geriatric Assessment (CGA), gait speed, hand grip strength or others were considered **non-specific criteria** because they measure parameters that might suggest frailty. Still, they were not explicitly created to diagnose or score the severity of frailty [1, 6, 11, 12]. REDCap data were extracted and analyzed using Sigma Plot 12.0. Summary statistics are presented using absolute and relative (percentage) values, mean and standard deviation (µ ± SD), or median and interquartile range (Q2-Q3) for parametric and non-parametric data accordingly.

Using any tool or specific tool to assess frailty and confidence in managing frailty was evaluated as a dichotomous outcome and compared using Chi-square or Mann-Whitney U tests. Factors identified as potentially crucial in univariate analyses were entered stepwise into a multivariate logistic regression model to determine the association between non-dependent variables. If multicollinearity was detected (VIF > 2.0) between two variables, the one with the lower P value at univariate analysis was included in the multivariate model. For all analyses, significance was set at 5%.

## Results

### Participant characteristics

Two hundred and seventy-one questionnaires were analyzed. Participants were employed in publicly funded organizations (120; 48%), private practice (78; 29%) or a mixture of both (60; 22.1%). Four participants did not respond to this question. The general characteristics, profession, and care sector of the participants are shown in Table 1.

Two hundred and twenty-eight (84%) of the participants lived in the Southeast region of Brazil, with the majority (219; 80%) coming from São Paulo state. However, 43 (16%) respondents covered all other Brazilian areas. Participants had graduated for a median (IQR) of 14 (5-20) years, with a median (IQR) of 10 (4-18) years spent working with older people. The percentage of older adults as a proportion of all patients seen in daily clinical practice varied considerably, with a median (IQR) of 50 (10–80) %.

**Table 1:** General characteristics, profession, and care sector of Brazilian health professionals participating in a study on identifying and managing frailty in daily practice (*n* = 271)

Female sex, *N* (%)	203.0 (76.0)
White, N (%)	214.0 (79.0)
Age between 25 and 49 years old, N (%)	187.0 (69.0)
**Professions**	
Medical doctors	112.0 (41.0)
Nurses, N (%)	34.0 (13.0)
Physiotherapists, N (%)	32.0 (12.0)
Dietitians, N (%)	31.0 (11.0)
Gerontologists, N (%)	18.0 (7.0)
Other healthcare professionals *, N (%)	44.0 (16.0)
**Care sector**	
Primary care, N (%)	65.0 (24.0)
Secondary care, N (%)	93.0 (34.0)
Tertiary care, N (%)	87.0 (32.0)
Community services, N (%)	24.0 (9.0)
Participants did not answer, N (%)	2.0 (0.74)

**Other healthcare professionals**: Dentist, psychologist, older people caregiver, health educator, nursing assistant, pharmacist, health technologist and teacher

### Frailty Assessment

**Fig. 1:**
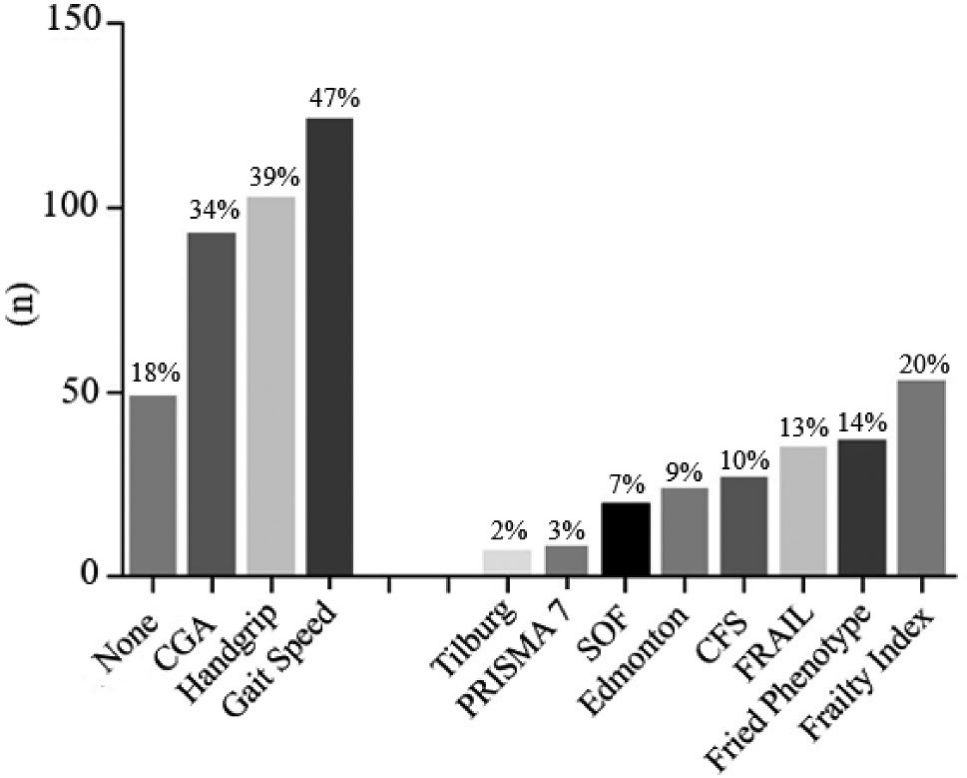
How the Brazilian Health Care Professionals assessed frailty; SOF: Study of Fracture; CFS: Clinical Frailty Scale; FRAIL Scale. Number of professionals who did not select any tool (none), who used non-specific (CGA, handgrip, gait speed) or specific criteria (Tilburg, PRISMA-7, SOF, Edmonton, CFS, FRAIL, Fried Phenotype, Frailty Index)

**Fig. 2:**
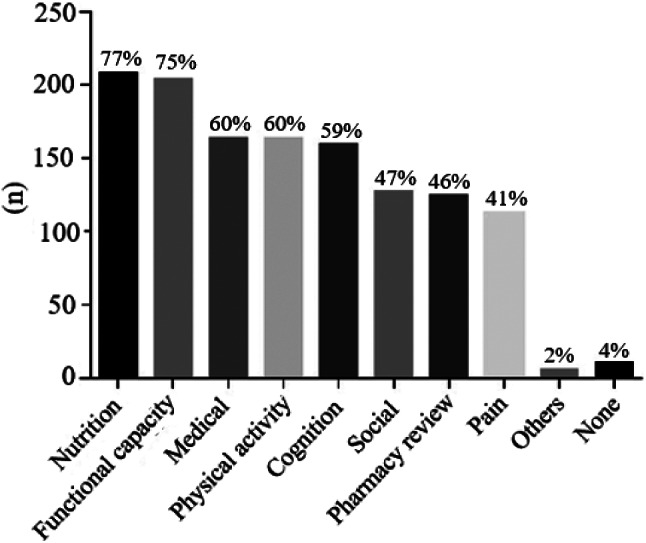
Number and frequency of professionals who suggested performing other assessments (nutritional, functional capacity, medical assessments, etc.) after identifying that a person lives with frailty

Two hundred and sixty-two (97%) respondents stated assessing frailty in routine clinical practice with older adults was important. Considering all 271 responses, 208 (76%) estimated frailty routinely; 56 (21%) responded that they did not evaluate frailty routinely, and, from these, 14 (5% of the total) could not define frailty. However, there needed to be more consistency between responses to the questions about assessing frailty and the criteria used. Twenty-eight participants responded that they never considered frailty but reported using one or more non-specific criteria for frailty assessment. In most instances, this relates to using criteria, such as handgrip strength or gait speed, for indications other than frailty. In addition, thirteen participants responded by saying that they assessed frailty but did not use a tool, reporting that frailty assessment was done “during clinical assistance, by observation, or by conversation and others.” Fig. 1 summarises these findings, showing that 48 (18%) participants selected none, and 223 (82%) used at least one specific or generic tool to measure frailty. After identification of frailty, 95% of participants suggested that further, more detailed assessments should be performed – many of which comprised components of CGA (Fig. 2).

### What factors explain the use of any tool or a specific tool?

Table 2 shows that participants who reported using any tool to assess frailty had fewer years of experience after graduation and fewer years of working with older people, a higher percentage of older people seen in daily practice as a proportion of all patients and were more likely to have received training around frailty. All the gerontologists reported using a frailty tool, while the lowest level of use was seen in the dietitian group.

**Table 2:** Univariate analyses associated with the assessment, confidence, and plan to manage Frailty among healthcare professionals in Brazil, 2020–2021

Assessment						
	Use of any tool			Use of any specific criteria		
	Yes *n* = 223	No *n* = 48	*p*-value	Yes *n* = 100	No *n* = 171	*p*-value
% of older people routinely assisted, median (range)	50.0 (15.0–80.0)	30.0 (9.00–60.0)	0.008	60.0 (18.7–90.0)	50.0 (10.0–70.0)	0.010
Years of experience, median (range)	12.0 (5.00–20.0)	18.5 (12.0-24.5)	0.001	14.0 (6.0–20.0)	13.0 (5.0–20.0)	0.580
Years of experience in older people assistance, median (range)	10.0 (3.00–17.0)	14.0 (8.00–22.0)	0.003	11.5 (5.7–20.0)	10.0 (3.0-17.5)	0.080
Trained before assessing frailty, % (n)	98.0 (107)	2.00 (2.00)	0.006	60.0 (65.0)	40.0(44.0)	< 0.001
Health Care Professionals						
Doctors, % (n)	83.0 (93.0)	17.0 (7.0)	0.006	48.0 (54.0)	52.0 (58.0)	0.002
Gerontologists, % (n)	100 (18.0)	0.00 (0.00)		44.0 (8.00)	56.0 (10.0)	
Physiotherapist, % (n)	94.0 (30.0)	6.00 (2.00)		44.0 (14.0)	56.0 (18.0)	
Nurses, % (n)	91.0 (30.0)	9.00 (3.00)		30.0 (10.0)	70.0 (23.0)	
Dietitians, % (n)	71.0 (22.00)	29.0 (9.00)		13.0 (4.00)	87.0 (27.0)	
Others, % (n)	70.0 (30.0)	30.0 ( 13.0)		23.0 (10.0)	77.0 (33.0)	
**Management**						
	**Confidence to Manage**		p-value	**Having a Management Plan**		p-value
	**Yes** *N* = 114	**No** *N* = 148		**Yes** *N* = 106	**No** *N* = 156	
% of older people routinely assisted, median (range)	50.0 (13.0–90.0)	50.0 (10.0–70.0)	0.104	60.0 (19.0–90.0)	45.0 (10.0–70.0)	< 0.001
Years of experience, median (range)	14.0 (7.00–20.0)	13.0 (4.00–20.0)	0.131	14.0 (6.00–20.0)	13.0 (5.00–20.0)	0.353
Years of experience in older people assistance, median (range)	11.0 (5.00–19.0)	10.0 (3.0–18.00)	0.067	11.0 (5.00–20.0)	10.0 (3.00–18.0)	0.304
Trained before managing frailty, % (n)	85.0 (58.0)	15.0 (10.0)	< 0.001	76.0 (52.0)	24.0 (16.0)	< 0.001
Health Care Professionals						
Doctors, % (n)	44.0 (48.0)	56.0 (62.0)	0.022	43.0 (48.0)	57.0 (63.0)	0.043
Gerontologists, % (n)	44.0 (8.00)	56.0 (10.0)		44.0 (8.00)	56.0 (10.0)	
Physiotherapist, % (n)	70.0 (21.0)	30.0 (9.00)		54.0 (15.0)	46.0 (13.0)	
Nurses, % (n)	44.00 (14.0)	56.0 (18.0)		48.0 (16.0)	52.0 (17.0)	
Dietitians, % (n)	26.0 (8.00)	74.0 (23.0)		16.0 (5.00)	84.0 (26.0)	
Others, % (n)	37.0 (15.0)	63.0 (25.0)		35.0 (14.0)	65.0 (26.0)	

Logistic regression confirmed that formal training increased the chance of assessing frailty by 15.6 (3.62–67.2) times and that the probability of formally assessing frailty was reduced by 5% for each year following graduation (Table 3, Model 1). The HCP category and the number of older adults seen as a proportion of all patients did not influence tool usage in the multivariate model. The model did not include years of work with older people because it showed multicollinearity with years of work after graduation.

**Table 3:** Multivariate logistic models used to evaluate the factors associated with the use of any tool or specific ones to assess frailty among healthcare professionals in Brazil, 2020–2021

**Use of any tool**			
	OR	95% CI	*p*-value
**Model 1**			
Years of experience *	0.95	0.92–0.98	0.01
% of older people routinely assisted	1.00	0.99–1.01	0.29
Professions	0.91	0.82–1.01	0.11
Previous training to assess	15.6	3.62–67.2	< 0.001
**Use of a specific tool**			
**Model 2**			
Years of experience in older people assistance	1.03	1.00-1.06	0.05
% of older people routinely assisted	1.00	0.99–1.01	0.23
Professions*	0.87	0.79–0.96	0.005
Previous training to assess	4.96	2.80–8.80	< 0.001
Model 3 to 7 **			
Doctor (Model 3)	2.05	1.14–3.66	0.01
Gerontologist (Model 4)	1.06	0.36–3.12	0.92
Physiotherapist (Model 5)	1.76	0.75–4.10	0.19
Nurse (Model 6)	0.63	0.25–1.55	0.31
Dietitian (Model 7)	0.29	0.09–0.91	0.03
**Being confident in managing frailty**			
**Model 8**			
Years of experience in older people assistance	1.02	0.99–1.06	0.15
% of older people routinely assisted	0.99	0.99–1.08	0.81
Professions	0.95	0.87–1.05	0.35
Previous training to assess	3.99	2.03–7.81	< 0.001
Previous training to manage	7.54	3.21–17.7	< 0.001
**Having a plan**			
**Model 9**			
Years of experience in older people assistance	1.01	0.98–1.05	0.32
% of older people routinely assisted	1.01	1.00-1.02	0.01
Professions	0.95	0.86–1.04	0.27
Previous training to assess	2.70	1.38–5.26	0.003
Previous training to manage	4.72	2.19–10.20	< 0.001

*professions were coded in numbers, so model 2 can only show the influence of occupations in the model. ** Models 3 to 7 were built considering, for example, being a doctor or not, and the same for the ‘Other professions. These models (3 to 7) were adjusted by years of experience assisting older people, % of older people daily assisted, and previous training (data not shown)

Previous training and professional categories were confirmed to be associated with using specific tools (Table 3, Model 2). Therefore, other models were built using the professional category as a dichotomous variable – which showed that being a doctor increases the chance of using specific criteria by 2.05 times (Table 3, Model 3) and being a dietitian decreased the likelihood by 3.44 times (Table 3, model 7). Other professional types were not associated with specific frailty tool use.

Assessing frailty using any tool, or specific ones, was similar among settings. For primary care professionals, 51 (78%) of HCPs used any tool, and 20 (31%) used specific criteria. The use of any tool was reported by 78 (84%) of secondary care and 73 (84%) of tertiary care respondents, respectively. The use of a specific tool was reported by 32 (34%) of secondary care and 38 (47%) of tertiary care respondents, respectively.

### Frailty management

Considering frailty management, after screening and assessment, 114 (42%) respondents said they felt confident managing frailty, and 68 (25%) reported having had formal training in frailty management. Respondents were divided between the 210 (77%) respondents who referred to geriatricians for ongoing frailty management and the 106 (39%) who felt competent to formulate their management plans (with some overlap between these groups demonstrated by those who both established their management plan in some instances and referred to geriatricians when care became more complex). Seventy-seven (28%) included medical and multidisciplinary therapies in their management plans, but only 31 (11%) routinely included end-of-life care, and 19 (7%) included urgent care.

Only 33 (12%) respondents had access to systems to share information about patient care between professionals, and 197 (75%) did not have, or did not know about, pathways to refer people living with frailty for further clinical follow-up. One hundred (37%) said their community had activities, such as entertainment, gymnastics, prayer groups, and crafts, for older people with frailty.

### What factors explain confidence and having a plan to manage frailty?

Confidence in managing frailty syndromes did not differ according to the percentage of older people as a proportion of all patients seen daily, as well as total years of experience or experience in the care of older people. However, HCPs who saw more older adults as a proportion of their overall patient load were more likely to be confident in establishing a management plan (*p* < 0.001). Training in the identification and management of frailty was associated with greater confidence. In the univariate analysis, confidence in managing frailty syndrome differed between different categories of HCP (*p* = 0.02).

Amongst those confident of establishing a management plan for frailty, the highest confidence was seen between physiotherapists, and the lowest amongst dietitians, as show in Table 2. However, the multivariate logistic regression (Table 3, Model 8) showed that training to assess and manage frailty was the main predictor of confidence in managing frailty.

The number of respondents who felt confident in establishing a management plan for frailty differed by category of HCP (*p* = 0.04). It was again highest in physiotherapists, followed by nurses, doctors, gerontologists, and dietitians, as presented in Table 2.

Both training to assess and manage and the percentage of older people as a proportion of the overall caseload influenced confidence to implement a plan for older people with frailty, as presented in Table 3, model 9.

## Discussion

The present online survey evaluated how Brazilian HCPs assess and manage frailty. The assessment methods were heterogeneous among different groups of professions, predominately using non-specific criteria. Training contributed to frailty assessment, use of specific criteria, confidence, and having a management plan.

All these facets can be explained, at least in part, by the fact that Brazil’s geriatric and gerontology areas are relatively new. The National Health Policy for Older People was created in 2006, with a lack of definition of frailty and placing it as synonymous with disability [13]. In addition, the Brazilian Society for Geriatrics and Gerontology completed 60 years of existence in 2022, but in 2010, there were only 51 places for medical residency in geriatrics [14]. National policy is relatively recent, and the available body of specialists to drive forward training and knowledge around the care of older people is small and slowly growing. Frailty care in Brazil is still in a relatively immature state – this perhaps explains why those professionals who trained more recently were more likely to assess for frailty and to be confident managing it. Those in practice for extended time need updating to keep pace with emerging evidence and practice in the field.

Brazil is experiencing rapid population aging. The country will experience a 30–45% increase in the population over 60 in the next 10–15 years [15]. Identifying older adults at risk of developing frailty is vital to preventing and managing geriatric conditions [16, 17]. Due to differences in frailty definition and the many specific criteria to evaluate it, frailty assessment can vary between settings and contexts [2, 18]. It is perhaps not surprising that there are gaps in frailty identification and management in clinical practice. From one side, gerontologists and geriatricians tended to use the most evidence-based approaches to frailty assessment and management, but approaches were less focused in other staff groups. Indeed, the most used criterion to assess frailty in the present survey was gait speed (45%), a simple and non-specific approach. It is worth noting that Clegg et al. have published a systematic review where gait speed had a high sensitivity, for identifying frailty against the standard criteria. However, the low specificity reduced the accuracy of gait speed as a single way to assess frailty [19]. One important finding was that hand grip strength was mentioned more commonly than the Fried Phenotype. It would be quite easy to progress on to measure a full Fried phenotype given that the availability of a hand grip dynamometer is the most common barrier to doing so. Lack of training impaired may explain the widespread failure to do so. The third most mentioned criterion was the CGA, however only 1/3 of the participants used it. The possible reasons are that this assessment is more time-consuming, the majority of the participants are not specialists and don’t assist many older people in their routines. In addition, even applying what the guides created by the Ministry of Health for primary care address for frailty diagnosis and management, the assessment of frailty without training might be challenging [20]. 

CGA has a lot to do with having the correct care systems and processes in place and fostering competencies in multidisciplinaryteamwork and communication, setting specific and measurable treatment goals and iterative care management over time. Training people to deliver CGA is not straightforward, although increasingly nuanced materials are available online thatmay require some adaptation for implementation in Brazil [21–23]. These findings, added to the fact that non-specific tools are quite common, raise the need for further discussions about the role of more straightforward non-specific ways of detecting frailty. Nevertheless, even amongst those professionals who were confident to put in place management plans for frailty, the lack of attention to the whole spectrum of frailty care – particularly palliative and urgent care – suggests that more work is needed to establish consistent and systematic approaches to comprehensive geriatric assessment – at least amongst the professionals studied.

Even whilst recognizing the importance of frailty assessment, some HCP respondents did not evaluate frailty routinely and could not define this syndrome. This finding mirrors another study conducted in Brazil, that evaluated only professionals working in primary care, mainly nurses (63%), finding limited knowledge concerning frailty. When asked to describe what they understood to be the frailty syndrome in older adults, just 13% of the participants in that study gave a correct answer [7]. 

There is some work to be done around education, training, and guidance for professionals. This could be impactful, given the evidence gathered here about the importance of specific training in shaping responses to frailty management. This has been demonstrated in other countries, such as Australia, where training has helped to surmount barriers to implementing frailty assessments [24]. 

The strengths of this study relate to the comprehensive and structured approach to questionnaire development and analysis, enabling multiple facets of frailty assessment and management to be understood. A further strength relates to the snowball sampling approach that enabled a wide range of HCPs from all regions of Brazil to be responded to. The primary limitations relate to the small sample of 271 respondents and the potential for bias, given that early respondents were likely to be enthusiasts for frailty, and would be more likely to snowball on to colleagues who were similarly engaged with the frailty agenda. With that, it may be that the proportion of professionals who do not feel confident in assessing or who do not identify frailty is greater than found in our results. Additionally, although the questionnaire was sent throughout Brazil and we received responses from several different states, the majority of responses were concentrated in the Southeast region of the country. Another important point to highlight as a limitation is that most participants worked in secondary and tertiary health care, which can enable access to equipment for assessing frailty, such as handgrip. These findings are likely to present an excessively popular view of how Brazilian HCPs think about and approach frailty. Given this, the signals detected here provide an essential insight that can inform work to better educate and support the workforce across multiple professional groups and healthcare sectors to deliver frailty-attuned care going forward.

## Conclusion

Frailty identification, confidence in management, and confidence to establish a management plan differed among Brazilian professionals by professional grouping. However, multivariate analysis found training to be the best overall predictor of being able to assess for and manage frailty. Our evidence strongly supports the need for agreement on standardized screening criteria and management plans that can be used across all professions where contact with frail older people is made. Attention to training at one or both undergraduate levels – to establish gold standard practice in new and postgraduate professionals – to address the knowledge deficit in those with more excellent experience- is essential. Focussing on these should be a national priority.

## Data Availability

Anonymized data may be made available for research by contacting the corresponding author. Any additional information or data required may be obtained from the corresponding author by email.
